# Incidence of Surgical Reintervention for Benign Prostatic Hyperplasia Following Prostatic Urethral Lift, Transurethral Resection of the Prostate, and Photoselective Vaporization of the Prostate: A TriNetX Analysis

**DOI:** 10.1016/j.euros.2023.11.009

**Published:** 2023-12-23

**Authors:** Jacob H. Feiertag, Jennifer A. Kane, Joseph Y. Clark

**Affiliations:** a Penn State College of Medicine, Hershey, PA, USA; b Department of Urology, Penn State Health Milton S. Hershey Medical Center, Hershey, PA, USA

**Keywords:** Benign prostatic hyperplasia, Prostatic neoplasm, Transurethral resection of prostate, Prostatectomy, Male urologic surgery, UroLift, Prostatic Urethral Lift, Reintervention

## Abstract

**Background:**

Prostatic urethral lift, or UroLift, has gained popularity as a treatment for lower urinary tract symptoms associated with benign prostatic hyperplasia (BPH). Surgical reintervention rates are a reliable indicator for treatment durability.

**Objective:**

The objective of this study was to utilize TriNetX, a third-party database, to investigate the incidence of surgical reintervention following UroLift, transurethral resection of the prostate (TURP), and photoselective vaporization of the prostate (PVP) procedures for BPH from 2015 to 2018.

**Design, setting, and participants:**

Male patients aged 18–100 yr diagnosed with BPH were identified in the TriNetX Diamond Network database between January 2015 and December 2018. Cohorts of individuals undergoing their first UroLift procedure were built using Current Procedural Terminology and International Classification of Diseases 10th Revision codes. TURP and PVP cohorts were built as comparison groups. The cohorts were then queried for subsequent BPH-related procedures.

**Outcome measurements and statistical analysis:**

Reprocedure rates were assessed and descriptive statistics were used.

**Results and limitations:**

The mean age at first-time UroLift was 70.1 ± 9.4 yr (*n* = 14 343). Cumulative reprocedure rates collected after first-time UroLift included 1 yr after UroLift (5.1%, *n* = 14 343) and 4 yr after UroLift (16.1%, *n* = 710), with an average annual increase of +3.6% per year following 1 yr after the procedure. Comparatively, TURP (*n* = 22 071) and PVP (*n* = 14 110) had 4-yr reprocedure rates of 7.5% and 7.8%, respectively, during the same timeframe. Limitations include a lack of clinical data and loss of follow-up data outside the Diamond Network.

**Conclusions:**

The reprocedure rate of UroLift at 4 yr is double the rate of TURP and PVP. In appropriately selected patients, UroLift might be a suitable option for those who desire symptomatic relief from BPH with minimal erectile and ejaculatory side effects. However, the risk of secondary surgical intervention should be considered when considering BPH treatments.

**Patient summary:**

We compared the reintervention rates of prostatic urethral lift (PUL), transurethral resection of the prostate (TURP), and photoselective vaporization of the prostate (PVP) using the TriNetX database, and have found that the highest reintervention rates were for PUL of 16% at 4 yr of follow-up, compared with about 8% for those who had TURP and PVP. Interestingly, the most common reintervention was the same operation at 1 yr. This has important implications when counseling patients about the durability of these various outlet procedures for BPH.

## Take Home Message

We compared the reintervention rates of prostatic urethral lift (PUL), transurethral resection of the prostate (TURP), and photoselective vaporization of the prostate (PVP) using the TriNetX database, and have found that the highest reintervention rates were for PUL of 16% at 4 yr of follow-up, compared with about 8% for TURP and PVP. Interestingly, the most common reintervention was the same operation at 1 yr. This has important implications when counseling patients about the durability of these various outlet procedures for benign prostatic hyperplasia.

## 1 Introduction

Benign prostatic hyperplasia (BPH) is one of the most prevalent genitourinary conditions experienced by aging men. BPH occurs in about half of men aged 60–79 yr and almost three-quarters of men aged 80 yr and older [1], [2]. Not all prostatic enlargement is pathologic, but the likelihood of benign prostatic obstruction (BPO) in the presence of bothersome lower urinary tract symptoms (LUTS) increases with age. One study found that 56% of men <80 yr of age, while 70% of men >80 yr of age, reported LUTS with BPH [3]. The management goals of BPH aim to reduce clinical symptoms as well as prevent or delay the progression. Watchful waiting with or without behavioral modification is typical practice. However, various pharmacologic and surgical treatments exist for patients with bothersome LUTS.

The predominant surgical intervention for BPO was transurethral resection of the prostate (TURP) until 2009 [4]. TURP procedures have been associated with a risk of complications including incontinence, strictures, erectile dysfunction, and loss of ejaculatory function [5], which has limited its widespread use. Photoselective vaporization of the prostate (PVP) is another surgical procedure that has increased in utilization. Some studies found PVP to be safer intraoperatively than TURP (including incidence rate of capsule perforation, transurethral resection syndrome, and lower transfusion requirement) but showed an increased risk for reoperation [6].

Minimally invasive procedures for BPO have increased in popularity within the past decade to manage LUTS while attempting to maintain a minimal side-effect profile. Prostatic urethral lift (PUL or UroLift) in particular has gained traction as a surgical procedure for BPH [7], [8]. PUL alters prostate anatomy via the placement of transprostatic suture implants, which pull the lumen of the prostatic urethra toward the capsule and widens the prostatic urethral lumen [9]. In some cases, the procedure can be done under local anesthesia/sedation, and in almost all cases, UroLift patients go home the same day. As per the 2021 guidelines, the American Urological Association (AUA) recommends that PUL be considered as a treatment option for prostate volumes of 30–80 ml with verified absence of an obstructive middle lobe [9]. In appropriately selected patients, studies have shown PUL to be effective in symptom management, including improvements of LUTS, International Prostate Symptom Score (IPSS), peak flow rates, patient satisfaction scores, and quality of life scores [8], [10], [11], [12], [13]. UroLift's nonablative and nonresective technique has been proved to be effective in preserving erectile and ejaculatory function [8], [10], [11], [12], [13], [14].

The minimally invasive nature of UroLift makes it an appropriate alternative option for men who are fearful of invasive treatment and whose primary goal is preserving ejaculatory function. However, it is possible that the use of these implants increases the need for surgical reintervention. Some studies have found that UroLift was not as effective in the improvement of symptoms as its invasive [14], [15] and minimally invasive counterparts [16]. An additional concern in the literature is the increased risk of failure of PUL in patients with middle lobe obstruction due to the potential for continued prostate tissue growth around the implants and intravesical prostatic protrusion. One study evaluated the safety and efficacy of PUL in this population, and posed that men with obstructive middle lobes and LUTS improved at least as well as those with lateral lobe obstruction following PUL [17]. However, this was a nonrandomized cohort study utilizing historic controls rather than a randomized control trial, limiting its applicability on a larger scale.

The rate at which a secondary procedure occurs, or the reprocedure rate, has been used by researchers to quantify procedure durability and efficacy [10], [18]. In the literature, there is variability in the reported surgical reintervention rates for patients after their first UroLift procedure. Previous studies have found that the UroLift reprocedure rates have been anywhere between 4.3% and 5.2% at 1 yr [18], [19], between 7.5% and 11.9% at 2 yr [19], [20], at 10.7% at 3 yr [21], and between 13.6% and 28.9% at 5 yr [10], [18]. The 5-yr results from the initial L.I.F.T. study demonstrated reprocedure rates that increase around 2–3% annually [10], while other meta-analyses published surgical reintervention rates closer to 6% per year [18].

The aim of this study was to utilize TriNetX, a third-party national healthcare database, to investigate the surgical reintervention rates of UroLift in relation to TURP and PVP from 2015 to 2018.

## 2 Patients and methods

The Diamond Network on the TriNetX database stores deidentified data on over 212 million patient entries collected via data aggregators from insurance claims clearinghouses. The network represents 99% of US health plans and includes data from electronic medical record vendors used in community-based primary and specialty care settings, medical claims from large clearinghouses, and pharmacy claims from Switches representing predominantly retail pharmacy transactions. Patients in the network include those who are living or may now be deceased, and cover a large variety of healthcare organizations in the USA. TriNetX, LLC is compliant with the Health Insurance Portability and Accountability Act (HIPAA). As this study used only deidentified patient records and did not involve the collection, use, or transmittal of individually identifiable data, this study was exempted from Institutional Review Board approval. All data collected from the TriNetX Diamond Network were current as of October 2023.

Utilizing the TriNetX Diamond Network, data were collected on male patients diagnosed with BPH who received their first UroLift, TURP, or PVP procedure during January 2015 to December 2018. Patients were identified in TriNetX using International Classification of Diseases 10th Revision (ICD-10) and Current Procedural Terminology (CPT) codes. Cohorts were built for a longitudinal analysis using relationships between CPT codes, ICD-10 codes, and the selected timeframe.

The ICD-10 codes used to identify male patients with BPH included BPH (N40) as well as other nondescript bladder dysfunction codes. CPT codes for the procedures under investigation included UroLift (52441 and 52442), TURP (52601), and PVP (52648). For an outcome analysis, additional CPT codes were used to identify the incidence of reprocedures following the initial procedure. The complete list of reintervention procedures analyzed includes UroLift, TURP, PVP, holmium laser enucleation of the prostate (HoLEP; 52649), transurethral needle ablation of the prostate (TUNA; 53852), Rezūm Water Vapor Therapy (Rezūm; 53854), Waterjet Ablation (0421T), transurethral microwave thermotherapy (TUMT; 53850), interstitial laser coagulation (52647), and transurethral incision of the prostate (TUIP; 52450).

UroLift, TURP, and PVP data collected within the Diamond Network spanned from January 2015 to December 2019. The timeframes for data collection were selected to allow for at least 1 full year of continuous data to be available for analysis. Thus, the time periods were chosen for the following years: 1 yr after the procedure (2015–2018), 2 yr after the procedure (2015–2017), 3 yr after the procedure (2015–2016), and 4 yr after the procedure (2015).

Analysis was restricted to the earliest procedure for each patient (index event), which included a diagnosis code related to BPH. Outcomes 1, 2, 3, and 4 yr after the initial procedure were further evaluated for reintervention with any of the ten listed procedures. Information collected from the cohorts and outcomes analysis was recorded.

## 3 Results

The average age at the index event of the first UroLift procedure was 70.1 ± 9.4 yr (*n* = 14 343). There were 103 patients who were 50 yr old or younger (0.7%), while 1012 patients were aged 90 yr and older (7.0%). The most common comorbidities at the time of the index event included hypertension (29.1%), diabetes mellitus (12.5%), chronic lower respiratory diseases (6.5%), and heart failure (2.0%).

**Fig. 1:**
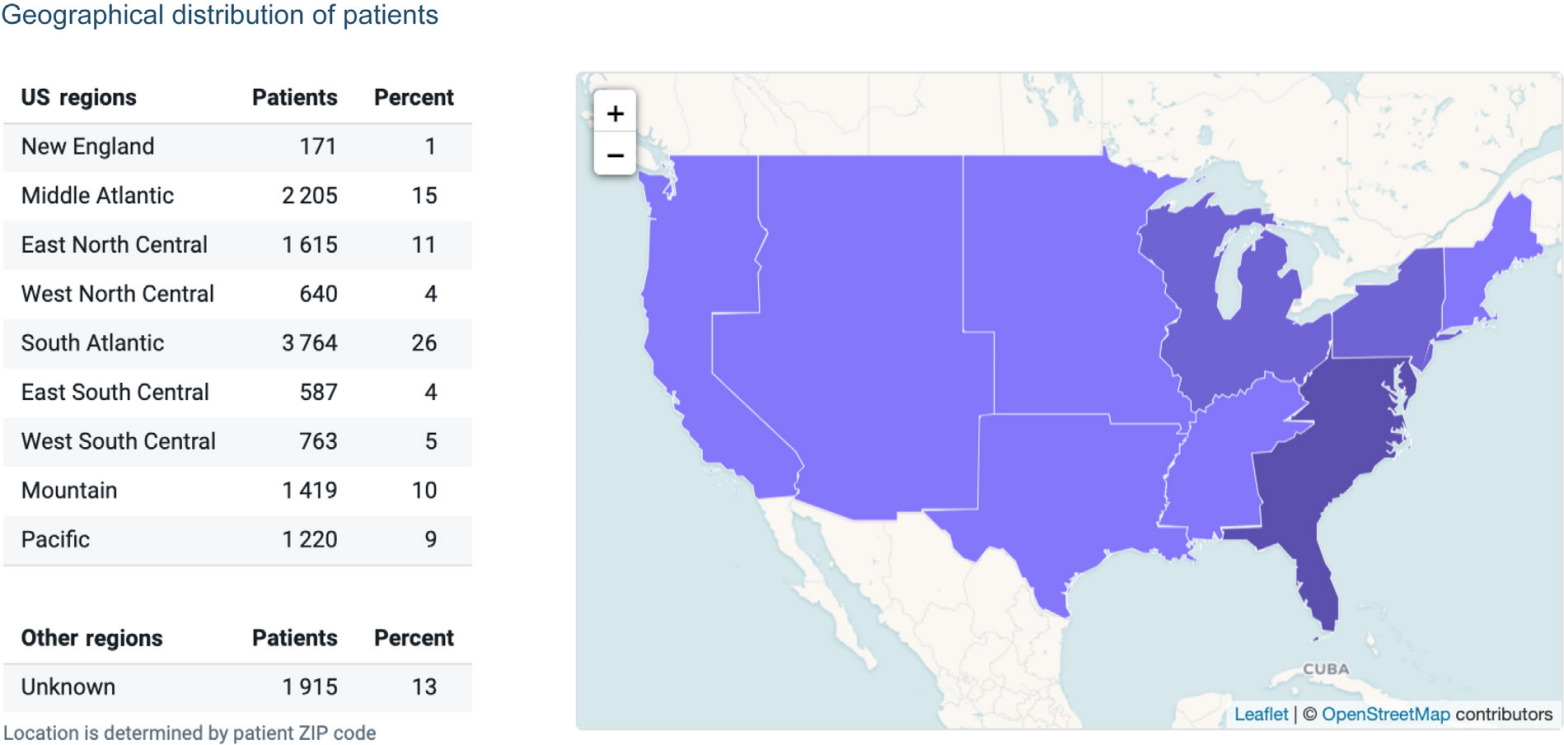
Geographical distribution of patients receiving their first UroLift procedure during 2015–2018 (*n* = 12 384). There were 1915 patients for whom geographical data were not provided.

Most patients’ racial demographic was unknown (62%). When racial demographics were reported, most UroLift patients were White (32%), followed by Black or African American (3%) and Asian (<1%). Most procedures available for analysis in the Diamond Network were performed in the South Atlantic region (26%), followed by the Middle Atlantic (15%) and East North Central (11%) regions (Fig. 1).

**Fig. 2:**
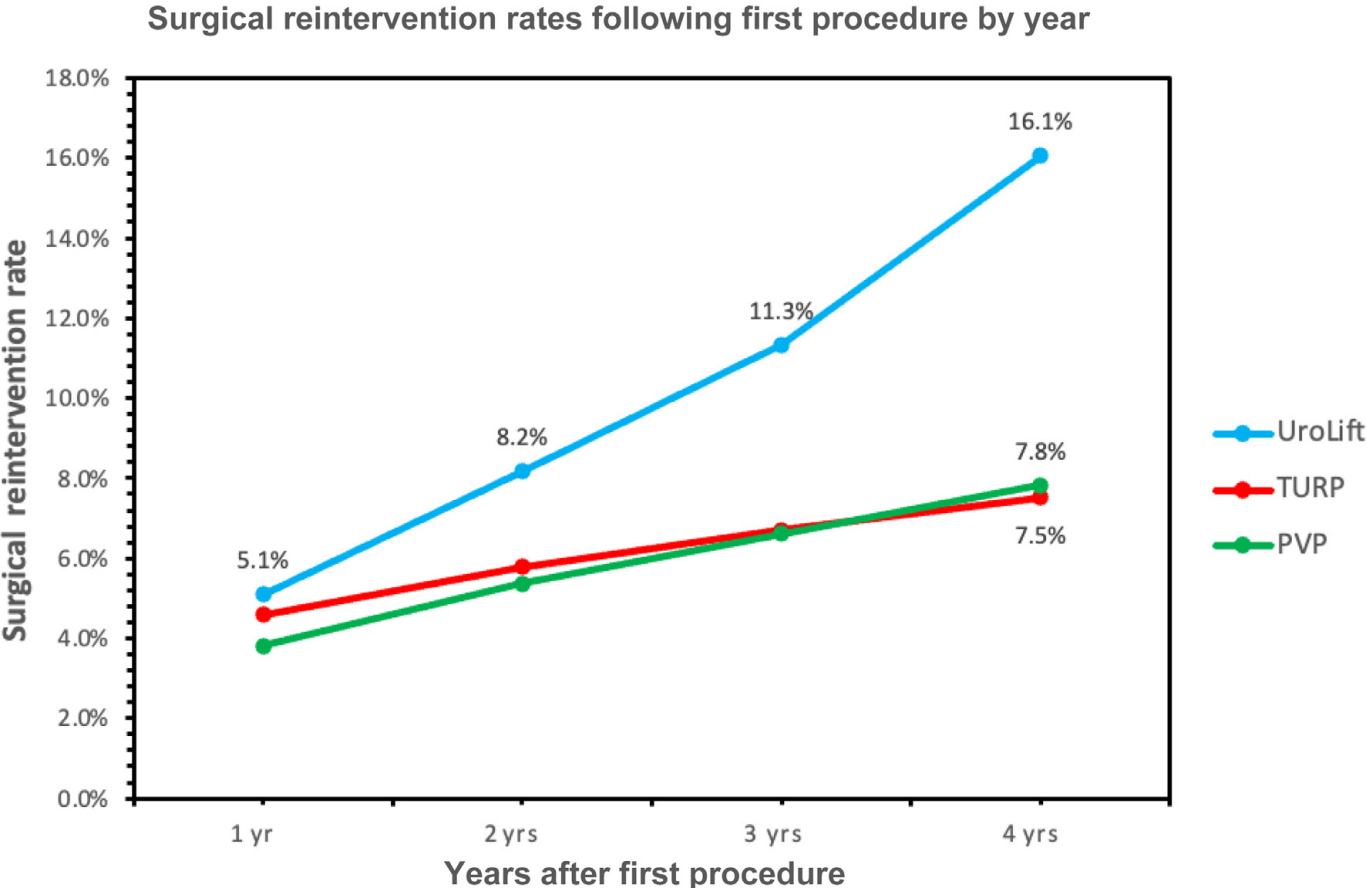
Cumulative surgical reintervention rate by designated yearly cohorts for UroLift, PVP, and TURP from 2015 to 2018. PVP = photoselective vaporization of the prostate; TURP = transurethral resection of the prostate.

**Fig. 3:**
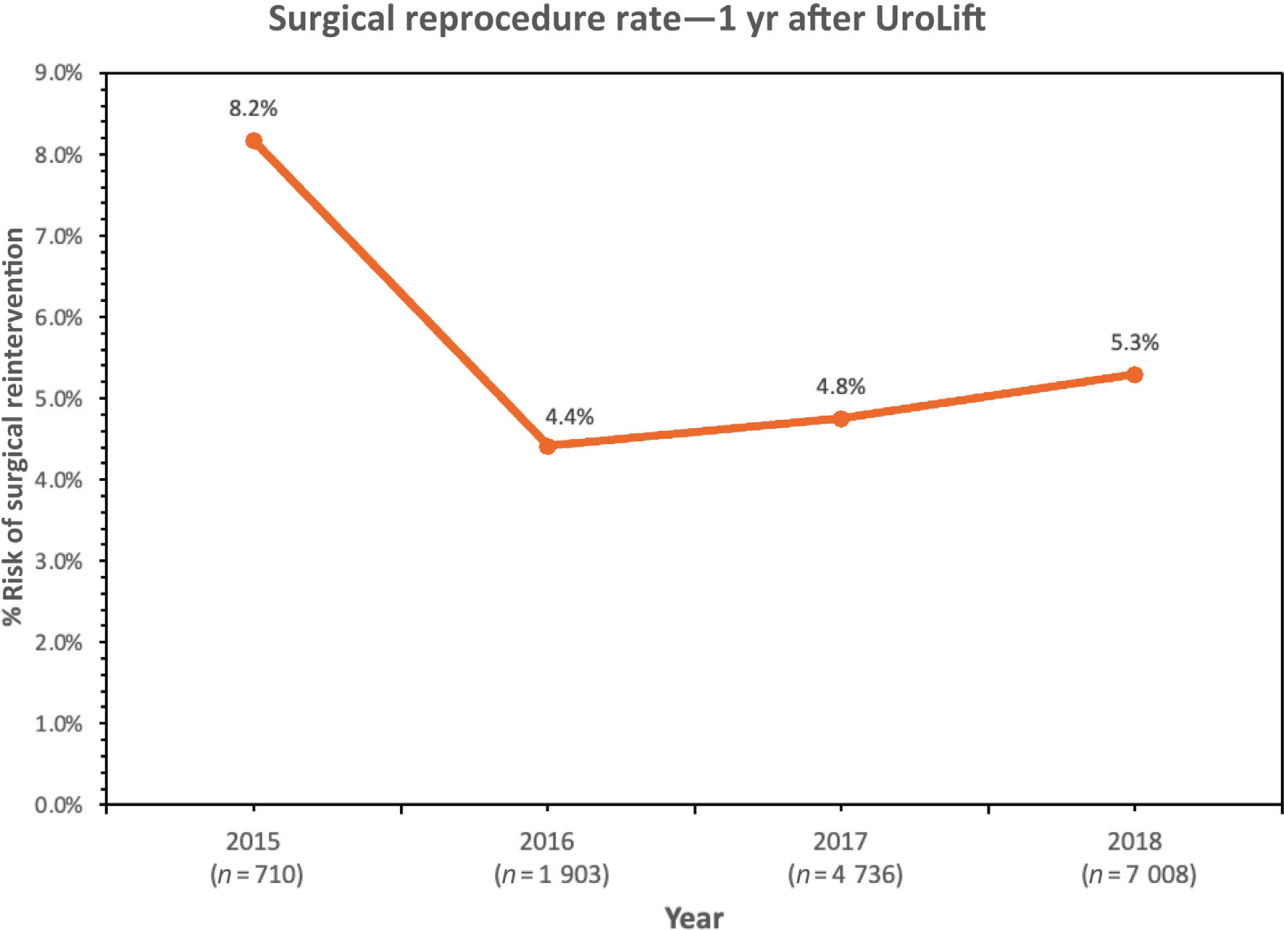
One year post-UroLift reprocedure rates from 2015 to 2018.

The total reintervention rate within 1 yr after the first UroLift was 5.1% (*n* = 14 343), followed by 8.2% (*n* = 7349) at 2 yr after UroLift, 11.3% (*n* = 2613) at 3 yr after UroLift, and 16.1% (*n* = 710) at 4 yr after UroLift (Fig. 2). The rate of reintervention following the first UroLift increased at an average rate of +3.6% per year after 1 yr following the procedure during 2015–2018. When looking at the reprocedure rates 1 yr after UroLift, there was an improvement in outcomes as time progressed. Starting in 2015, the reprocedure rate for UroLift was 8.2% at 1-yr follow-up. In the following years, there was a decrease—4.4% in 2016, 4.8% in 2017, and 5.3% in 2018 (Fig. 3).

Other procedures for BPH demonstrated similar reintervention rates at 1 yr, but lower rates at 4 yr. TURP demonstrated a reintervention rate of 4.6% at 1 yr (*n* = 92 425) and 7.5% at 4 yr (*n* = 22 071), while PVP demonstrated a reintervention rate of 3.8% (*n* = 51 439) at 1 yr and 7.8% (*n* = 14 110) at 4 yr. The annual reintervention rate increased by +1.0% for TURP and +1.3% for PVP from years 1 to 4 after the procedure (Fig. 2).

**Fig. 4:**
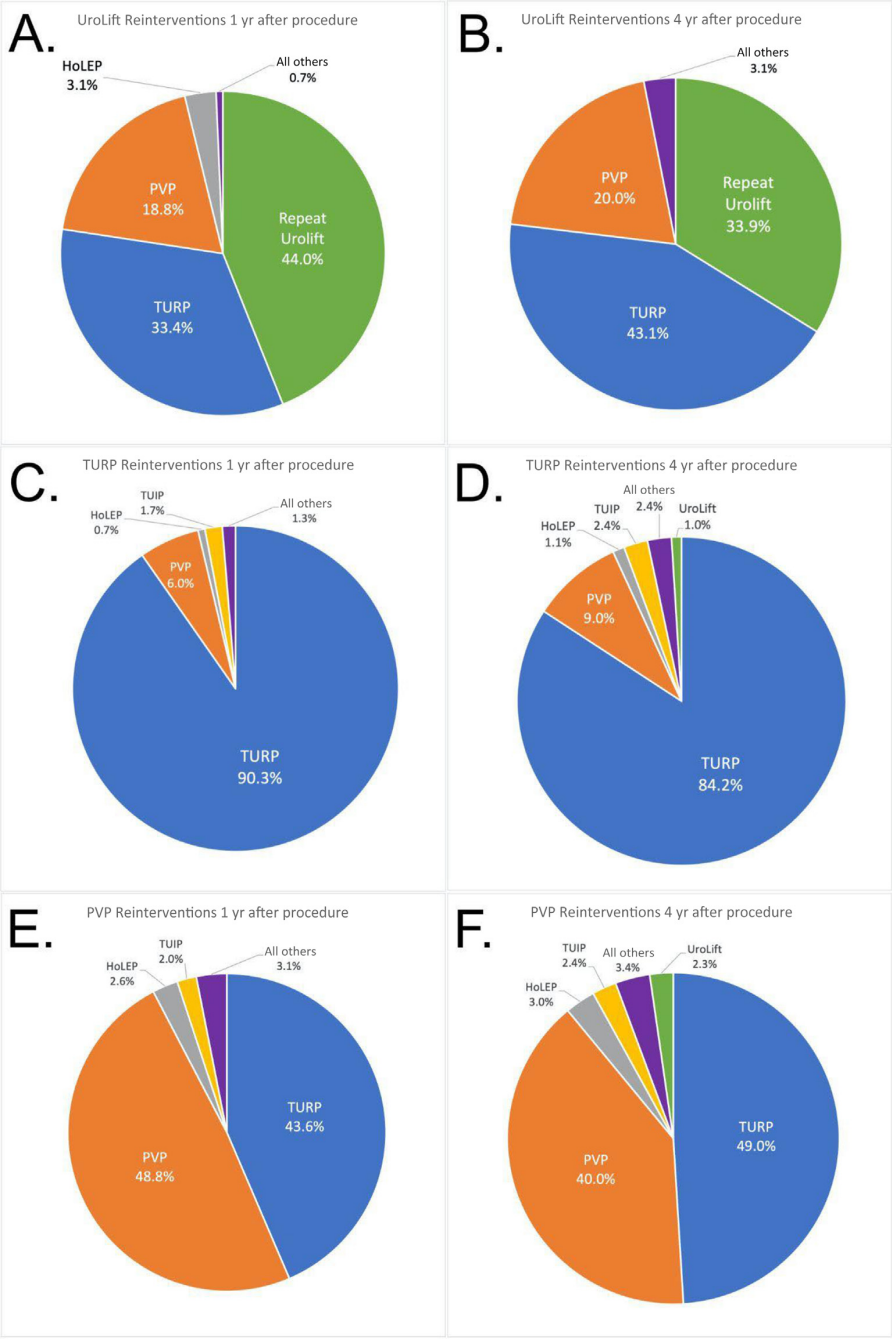
Distribution of the most common surgical reinterventions from 1 yr after the procedure to 4 yr after the procedure. Reinterventions for (A and B) UroLift, (C and D) TURP, and (E and F) PVP are included. HoLEP = holmium laser enucleation of the prostate; PVP = photoselective vaporization of the prostate; TUIP = transurethral incision of the prostate; TURP = transurethral resection of the prostate.

Types of interventions were tracked to identify which reprocedures were most common for each respective procedure. For the UroLift cohort, the most common reprocedures at 1 yr included repeat UroLift (44.0%), TURP (33.4%), PVP (18.8%), and HoLEP (2.6%; Fig. 4A), while at 4 yr, the most common reprocedure included TURP (43.1%), followed by repeat UroLift (33.9%) and PVP (20.0%; Fig. 4B).

The TURP cohorts demonstrated a bias to perform a second TURP. At the 1-yr mark, 90.3% of patients received a second TURP reprocedure, followed by PVP (6.0%) and TUIP (1.3%; Fig. 4C). For TURP patients at the 4-yr timeline, TURP remained the most common reprocedure (84.2%) followed by PVP (9.0%), TUIP (2.4%), HoLEP (1.1%), and UroLift (1.0%; Fig. 4D).

The PVP cohort at the 1-yr postprocedure time point showed that most patients received a second PVP (48.8%) followed by reintervention with TURP (43.6%), HoLEP (2.6%), and TUIP (2.0%; Fig. 4E), while the 4-yr postprocedure time point demonstrated that TURP was the most common procedure (49.0%), followed by repeat PVP (40.0%), HoLEP (3.0%), TUIP (2.4%), and UroLift (2.3%; Fig. 4F).

There were no instances of reintervention with Waterjet Ablation across all cohorts.

## 4 Discussion

UroLift is one of the several new minimally invasive surgical approaches to treat symptomatic BPH. The surgical reintervention rate following an initial procedure can be studied as an indicator of procedure durability for long-term management of BPO/LUTS.

This study found an average annual increase in the reprocedure rate for UroLift of +3.6% per year after 1 yr from the procedure during 2015–2018. Comparatively, this finding is higher than the annual reprocedure rate of 2–3% found in the L.I.F.T. 5-yr trial (*n* = 104) [10] and lower than a meta-analysis of 11 studies, which found the annual increase in the reintervention rate for PUL to be closer to 6% (*n* = 2106) [18]. Additionally, this study reported a cumulative reprocedure rate of 5.1% at 1 yr, 8.2% at 2 yr, 11.3% at 3 yr, and 16.1% at 4 yr after UroLift. Our research also found that the most frequent reprocedure after UroLift was a second UroLift at the 1-yr mark. By the 4th year, however, TURP emerged as the predominant choice. While there are no established guidelines for follow-up procedures after UroLift, patients might elect for additional implants to continue benefiting from UroLift's minimal side effects (such as preserving ejaculatory function) before considering more invasive procedures for definitive treatment. Additionally, it seems that urologists tend to offer what they are comfortable doing as a reintervention. As our data show, patients who had a reintervention at 1 yr after a UroLift, PVP, or TURP tended to have that same procedure as a reintervention.

The cumulative reintervention rate following UroLift was higher than that for TURP or PVP. When querying for reprocedures during the same time periods within TriNetX, the secondary intervention rate for UroLift was similar at 1 yr to that for TURP and PVP. However, TURP demonstrated a reprocedure rate of 7.5% after 4 yr (*n* = 22 071), while PVP demonstrated a reprocedure rate of 7.8% (*n* = 14 110), nearly half the reprocedure rate of UroLift at 4 yr. Other studies have found relatively similar results for TURP (12.9% at 5 yr [22]) and PVP (6.1–21.0% at 5 yr [22], [23], [24]). Additionally, this study showed that the UroLift annual reprocedure rate was nearly three times higher after the 1st year than that of the other procedures in the study (+3.6% per year for UroLift, +1.0% for TURP, and +1.3% for PVP).

The higher rate of reprocedures associated with UroLift could be attributed to a combination of factors. UroLift is a purely mechanical procedure, placing implants to retract the prostate tissue. Fundamentally, it does not remove tissue using energy such as the other outlet procedures, which may contribute to higher reprocedure rates. Furthermore, UroLift is a relatively new procedure—surgeons who are less experienced with UroLift may initially have higher rates of retreatment. Data from our study indicated that there was an initial learning curve, but reprocedure rates decreased from 2015 to 2018 after 1-yr follow-up. As urologists continue to refine their implantation technique, gain more experience, and improve their criteria for patient selection, we anticipate a reduction in reprocedure rates to a baseline.

Thermal ablative procedures, comparatively, have varying reliability in terms of reprocedure rates. Studies showed that TUMT is associated with reintervention rates of 19.4% at the 2.5-yr mark [25] and 19.8% at 3 yr [26]. TUNA’s efficacy declines in the long term with a rate of secondary treatment significantly higher than that of TURP (odds ratio of 7.44) [27], while another study found TUNA to have a reprocedure rate of 9.5% at 5 yr [28]. Of note, TUNA is not recommended for the treatment of BPH/LUTS as per the 2021 AUA guidelines [9].

Most patients prefer lower-risk management options with a desire for treatments that have fewer sexual side effects and are primarily effective at improving urgency, incontinence, and nocturia [29]. The nonablative nature of UroLift demonstrates some promise for patients looking for symptomatic management of BPH while maintaining a minimal sexual side-effect profile. The BPH6 trial randomized patients to UroLift and TURP, and showed that TURP was superior to UroLift in IPSS and Qmax improvements, but inferior to UroLift in terms of quality of recovery and ejaculatory function preservation [30]. Although objective urinary flow improvements and reprocedure rates are better with tissue ablation procedures such as TURP, patients who desire a quick recovery and preservation of ejaculatory function may opt for the UroLift procedure. Patient selection is a critical component for those electing for PUL therapy, as gland morphology, gland volume, or the presence of an obstructive middle lobe may negate the beneficial effects of the procedure. The surgical reprocedure data in this study can be integrated with findings from other research studies (such as the L.I.F.T. trial) to help assess UroLift’s value as a long- versus short-term surgical remedy for BPO/LUTS caused by BPH in those eligible for the procedure.

There are some limitations in the study design. The TriNetX database can be used only to extrapolate overall performance indicators (such as the occurrence of reprocedures) and does not allow for assessment of specific patient characteristics (such as the severity of symptoms, size of prostate enlargement, presence of median lobe, IPSS, uroflowmetry, number of implants placed, etc.) or surgeon expertise (surgical volume). Additionally, patient information on the TriNetX Diamond Network is compiled from the 92 servers that contribute to the database. These data may not represent all patients seeking BPH therapies. Additionally, if patients go outside these participating networks, their patient information will not be captured for analysis. Further analysis is needed to evaluate the continued effectiveness of LUTS management (measured via IPSSs, peak flow rates, and quality of life scores) as well as the longevity of treatment (through reintervention rates). Of note, this study did not control for the concomitant use of pharmacologic BPH treatments before or after UroLift. The creation of a washout cohort on TriNetX would have greatly reduced cohort populations considering that many men use pharmacologics in conjunction with surgical procedures for BPH. Reasons for reintervention (persistent symptoms, poor patient selection, encrustation of implants, etc.) were unable to be discerned due to limitations of the database.

## 5 Conclusions

The cumulative reprocedure rate for UroLift at 4 yr was twice that of TURP and PVP. The favorable side-effect profile could make UroLift a popular procedure for those who desire to preserve erectile and ejaculatory function, but the reprocedure rate should be considered when selecting a surgical treatment modality for men with BPH.

  ***Author contributions:*** Jacob H. Feiertag had full access to all the data in the study and takes responsibility for the integrity of the data and the accuracy of the data analysis.

  *Study concept and design*: Feiertag, Clark.

*Acquisition of data*: Feiertag, Kane.

*Analysis and interpretation of data*: Feiertag, Clark.

*Drafting of the manuscript*: Feiertag, Kane.

*Critical revision of the manuscript for important intellectual content*: Clark.

*Statistical analysis*: Feiertag.

*Obtaining funding*: None.

*Administrative, technical, or material support*: None.

*Supervision*: Clark.

*Other*: None.

  ***Financial disclosures:*** Jacob H. Feiertag certifies that all conflicts of interest, including specific financial interests and relationships and affiliations relevant to the subject matter or materials discussed in the manuscript (eg, employment/affiliation, grants or funding, consultancies, honoraria, stock ownership or options, expert testimony, royalties, or patents filed, received, or pending), are the following: None.

  ***Funding/Support and role of the sponsor:*** Access to the TriNetX database was provided by the Penn State Clinical and Translational Science Institute, which is supported by the National Center for Advancing Translational Sciences, National Institutes of Health, through Grant UL1 TR002014. The content is solely the responsibility of the authors and does not necessarily represent the official views of the NIH.
